# A Model-Based Approach to Trial-By-Trial P300 Amplitude Fluctuations

**DOI:** 10.3389/fnhum.2012.00359

**Published:** 2013-02-08

**Authors:** Antonio Kolossa, Tim Fingscheidt, Karl Wessel, Bruno Kopp

**Affiliations:** ^1^Institute for Communications Technology, Technische Universität BraunschweigBraunschweig, Germany; ^2^Cognitive Neurology, Technische Universität BraunschweigBraunschweig, Germany; ^3^Department of Neurology, Braunschweig HospitalBraunschweig, Germany; ^4^Department of Neurology, Hannover Medical SchoolHannover, Germany

**Keywords:** predictive surprise, Bayesian surprise, event-related brain potentials, P300, single trial EEG, digital filtering

## Abstract

It has long been recognized that the amplitude of the P300 component of event-related brain potentials is sensitive to the degree to which eliciting stimuli are surprising to the observers (Donchin, 1981). While Squires et al. (1976) showed and modeled dependencies of P300 amplitudes from observed stimuli on various time scales, Mars et al. (2008) proposed a computational model keeping track of stimulus probabilities on a long-term time scale. We suggest here a computational model which integrates prior information with short-term, long-term, and alternation-based experiential influences on P300 amplitude fluctuations. To evaluate the new model, we measured trial-by-trial P300 amplitude fluctuations in a simple two-choice response time task, and tested the computational models of trial-by-trial P300 amplitudes using Bayesian model evaluation. The results reveal that the new digital filtering (DIF) model provides a superior account of the trial-by-trial P300 amplitudes when compared to both Squires et al.’s (1976) model, and Mars et al.’s (2008) model. We show that the P300-generating system can be described as two parallel first-order infinite impulse response (IIR) low-pass filters and an additional fourth-order finite impulse response (FIR) high-pass filter. Implications of the acquired data are discussed with regard to the neurobiological distinction between short-term, long-term, and working memory as well as from the point of view of predictive coding models and Bayesian learning theories of cortical function.

## 1 Introduction

The notion of a Bayesian brain is increasingly recognized as providing a distinctive framework for investigating cognitive brain functions (Kersten et al., 2004; Knill and Pouget, 2004; Friston, 2005; Doya et al., 2007). Predictive coding theories of cortical function provide a possible route to the Bayesian brain (Friston, 2002). According to the predictive coding approach, constraints from higher levels of a cortical hierarchy provide contextual guidance to lower levels of processing, providing a theory of how bottom-up evidence is combined with top-down priors to compute the most likely interpretation of sensory data. Specifically, predictive coding theory proposes that an internal representation of the world generates predictions that are compared with stimulus-driven activity to calculate the residual error between the predicted and the actual information. The residual error is then used to update the internal representation so as to minimize the residual error imposed by future stimuli (Friston, 2002, 2005; Spratling, 2010).

The general scheme of predictive coding as a ubiquitous mode of cortical processing offers an instrumental framework for analyzing functional correlates of the P300 event-related brain potential (Sutton et al., 1965; Kopp, 2008). It has long been recognized that fluctuations in P300 amplitude reflect the degree of surprise related to the processing of attended, but unforeseeable sensory events. In particular (Donchin, 1981) argued that P300 amplitude is not crucially determined by the inherent attributes of eliciting events. Instead of that, he ascertained that “surprising events elicit a large P300 component” (p. 498). Squires et al. (1976) had presented a model of P300 amplitude fluctuations, based on the concept of expectancy, which was thought to be determined by three factors: “(i) the memory for event frequency within the prior stimulus sequence, (ii) the specific structure of the prior sequence, and (iii) the global probability of the event” (p. 1144).

More recently, Mars et al. (2008) proposed a computational model of processes underlying the generation of the P300 in which trial-by-trial fluctuations in P300 amplitudes were explained in terms of a Bayesian observer keeping track of the global probabilities of sensory events. The subjective estimates of statistical regularities in the environment were thought to depend crucially on the integration of sensory data over long periods of time. However, the adequacy of this Bayesian observer model is limited, because it cannot account appropriately for the well-documented effects of the recent stimulus sequence on P300 amplitudes (e.g., Squires et al., 1976; Leuthold and Sommer, 1993).

Here we tested these two state-of-the-art models against a newly developed computational model of trial-by-trial P300 amplitude fluctuations by Bayesian model selection (Kass and Raftery, 1995; Raftery, 1995). The new model assumes three additive digital filtering processes, thereby integrating aspects of both state-of-the-art models. Specifically, subjective estimates of statistical regularities in sensory data are kept at short-term and long-term decay time parameters. Further it implements an alternation term (as Squires et al., 1976) as well as uniform initial prior probabilities (as Mars et al., 2008). Our findings show that this new approach provides a superior account of parietally distributed trial-by-trial P300 amplitudes compared to these two state-of-the-art models.

## 2 Materials and Methods

### 2.1 Participants, experimental design, and data acquisition

Sixteen healthy participants [fourteen women, mean age: 20 years; age range 18–23 years; mean handedness (Oldfield, 1971): 74; handedness range −76–100], all with normal or corrected-to-normal visual acuity participated in the experiment. All were recruited from introductory courses at the Department of Psychology at the Technische Universität Braunschweig in return for course credit. Experimental procedures were approved by the local ethics committee and in accordance with the Declaration of Helsinki.

Participants performed a simple two-choice response time [RT] task without feedback about response accuracy in which all stimuli had equal behavioral relevance. This feature of the experimental design constitutes an important difference between this and the classical oddball paradigm (Ritter and Vaughan, 1969) in which participants usually discriminate between task-relevant (target) and irrelevant (standard) stimuli.

The experiment was realized using the Presentation^®^ software (Neurobehavioral Systems, Albany, CA, USA). Visual stimuli were presented one at a time for 100 ms each, with a stimulus presentation rate of *f_s_* = 2/3 Hz, i.e., one stimulus per 1.5 s. Stimuli were displayed at the center of a CRT monitor (FlexScan T766 19″; Eizo, Hakusan, Ishikawa, Japan) with a refresh rate of 100 Hz at a resolution of 1280 × 1024 pixels against a light gray background. Viewing distance amounted to 1.25 m. Two types of visual stimuli were presented: the stimulus event was either a red or a blue rectangle, each of which subtended approximately 2.75° × 2.25°.

Participants were required to respond to each stimulus with the previously associated button as quickly as possible but not at the expense of accuracy. They used the index finger of both hands (e.g., left button on response to the red rectangle, right button in response to the blue rectangle). Stimulus-response mapping (i.e., [red-left, blue-right] or [red-right, blue-left], respectively) was counterbalanced over participants.

Participants performed twelve blocks of *N* = 192 trials of the two-choice RT task. The probability of the occurrence of each stimulus event was manipulated between blocks such that the relative probabilities of events were either 0.5 for each event, across six consecutive blocks (1152 trials overall), or [0.3, 0.7], across the remaining six consecutive blocks (1152 trials overall). Stimulus-probability mapping was counterbalanced over participants (i.e., a stimulus color identified the rare (0.3) stimulus in fifty percent of the participants but the frequent stimulus (0.7) in the remaining participants).

The order of the probability manipulation was counterbalanced over participants (probability category [0.5, 0.5] prior to [0.3, 0.7] or vice versa) *who were not informed about these probabilities*. Participants were informed that the two different stimuli were randomly distributed across blocks. Between the blocks a break was scheduled, participants were free to initiate the subsequent block at their own pace.

A continuous electroencephalogram (EEG) was recorded using a QuickAmps-72 amplifier (Brain Products, Gilching, Germany) and the BrainVision Recorder^®^ Version 1.02 software (Brain Products, Gilching, Germany) from frontal (F7, F3, Fz, F4, F8), central (T7, C3, Cz, C4, T8), parietal (P7, P3, Pz, P4, P8), occipital (O1, O2), and mastoid (M1, M2) sites. Ag-AgCl EEG electrodes were used which were mounted on an EasyCap (EasyCap, Herrsching-Breitbrunn, Germany). Electrode impedance was kept below 10 kΩ. All EEG electrodes were referenced to average reference during the recording.

For each participant, the actual stimulus sequence of each probability category [0.5, 0.5], and [0.3, 0.7], respectively, was randomized only once in order to enhance the reliability of the sequential trial-by-trial P300 estimates (see below). Thus, each participant received solely one truly random arrangement of trials in each probability category. This arrangement was repeatedly presented across all six blocks of each probability category, unbeknownst to participants. In consequence, sequential P300 estimates could be averaged over the six sequence repetitions per probability category, thereby improving the notoriously low signal-to-noise ratio of single-trial EEG data. Task-related brain activity of a single trial is much more obscured by task-unrelated brain activity than is task-related activity averaged across trials (Blankertz et al., 2002).

Participants were informed about the problem of non-cerebral artifacts, and they were encouraged to reduce the occurrence of movement artifacts (Picton et al., 2000). Ocular artifacts were monitored by means of bipolar pairs of electrodes positioned at the sub and supraorbital ridges (vertical electrooculogram, vEOG) and at the external ocular canthi (horizontal electrooculogram, hEOG). The EEG and EOG channels were subject to a bandpass of 0.01–30 Hz and digitized at 250 Hz sampling rate.

Off-line analysis of the EEG data was performed by means of the BrainVision Analyzer^®^ Version 2.0.1 software (Brain Products, Gilching, Germany). Careful manual artifact rejection was performed before averaging to discard trials during which eye movements, or any other non-cerebral artifact except blinks, had occurred. Deflections in the averaged EOG waveforms were small indicating that fixation was well maintained in those trials that survived the manual artifact rejection process. Semi-automatic blink detection and the application of an established method for blink artifact removal were employed for blink correction (Gratton et al., 1983). A digital high-pass filter was applied to the data (0.75 Hz cutoff frequency, 48 db/oct) in order to eliminate low-frequency variations in the EEG signal which were associated with the occasional occurrence of electro-dermal artifacts.

The EEG was then divided into epochs of 1000 ms duration, starting 100 ms before stimulus onset. Epochs were corrected using the interval [−100, 0 ms] before stimulus presentation as the baseline. As a start, event-related potential (ERP) waveforms were created (Luck, 2005). ERP waveforms were calculated as trial averages for each participant and for each event probability [i.e., 0.5, 0.3, 0.7], with the exception that those trials in which the participant selected the wrong behavioral response were excluded from averaging.

Thereafter, trial-by-trial P300s were estimated from the EEG data at electrode Pz, where this ERP component is traditionally reported to be maximal (Duncan-Johnson and Donchin, 1977). To estimate trial-by-trial P300 amplitudes, for each participant, the time point at which the averaged P300 waveforms at Pz were modulated maximally by relative stimulus frequency in the [0.3, 0.7] probability category was determined (*M* = 344 ms, *SD* = 48 ms; range 280–464 ms). Identifying the P300 in single trials is a notoriously difficult problem, due to the low signal-to-noise ratio of single-trial EEG data (Blankertz et al., 2002). In our study, for each event probability, trial-by-trial P300 estimates were extracted over a temporal window of ±60 ms around the individual time point of maximal modulation (Barceló et al., 2008), thereby completely ignoring latency variability across single trials (Luck, 2005). Albeit this drawback of the method, it was nevertheless chosen in order (1) to keep the testing environment as similar as possible to the procedures employed by Mars et al. (2008), and (2) to improve the reliability of trial-by-trial amplitude measures, in comparison to peak detection measures, akin to previous studies (Debener et al., 2005).

### 2.2 Conventional data analysis

Trial-by-trial P300 estimates, RTs, and error rates were averaged according to the three event probabilities [i.e., 0.3, 0.5, 0.7]. In the [0.5, 0.5] probability category, trial-by-trial P300 estimates were additionally averaged according to eight third-order stimulus sequences (denoted as *aaaa*, *baaa*, *abaa*, *aaba*, *bbaa*, *abba*, *baba*, *bbba*), four second-order stimulus sequences (*aaa*, *baa*, *aba*, *bba*), and two first-order sequences (*aa*, *ba*), with up to four consecutive trials (*xxxx*) = (trial *n* − 3, trial *n* − 2, trial *n* − 1, trial *n* = eliciting event). Please note that the symbol *a* simply denotes one of the two possible stimulus events while symbol *b* signifies the other one in this notation. For example, if *a* signifies the red rectangle, then *b* signifies the blue rectangle (and vice versa). In the [0.5, 0.5] probability category, sequential analysis could be collapsed across the two possible stimulus events since both stimuli were equally probable and task-relevant. The same kind of sequential analysis was performed in the [0.3, 0.7] probability category. However, in this experimental condition, *a* consistently denoted the rare stimulus in the [0.3, 0.7] probability category, whereas *b* signified the frequent stimulus in the [0.3, 0.7] probability category. We refrained from analyzing the eight third-order stimulus sequences in the [0.3, 0.7] probability category in order to obtain a sufficient number of trials entering the sequential P300 estimates. Thus, trial-by-trial P300 estimates were solely averaged according to eight second-order stimulus sequences (*aaa*, *baa*, *aba*, *bba*, *bbb*, *abb*, *bab*, *aab*) and four first-order sequences (*aa*, *ba*, *bb*, *ab*) in the [0.3, 0.7] probability category, separately for rare and frequent stimuli.

Individual medians of trial-by-trial P300 estimates, RTs, and error rates over the three event probabilities [i.e., 0.3, 0.5, 0.7] as well as the sequential P300 estimates, generated as described above, were submitted to repeated measures analysis of variance (ANOVAs), using the Greenhouse–Geisser correction. The results of the univariate tests are provided, using a format which gives the uncorrected degrees of freedom, and ε in order to compensate for violations of sphericity or equal covariance among all pairs of levels of the repeated measures (Picton et al., 2000). A measure of effect size, $\eta_{p}^{2}$ (partial eta squared), is also provided.

### 2.3 State of the art models

Let us call

(1) $$P_{k}\left(n\right)=P\left(s\left(n\right)=k|{\text{s}}_{1}^{n-1}\right)\;\text{with }k\in \left\{1,\ldots ,K\right\}$$

an *estimated subjective probability* (henceforth simply called *subjective probability*) that event *k* ∈ {1, …, *K*} on trial *n* ∈ {1, …, *N*} will be observed, given a sequence ${\text{s}}_{1}^{n-1}=\left(s\left(1\right),s\left(2\right),\ldots ,s\left(n\;-\;1\right)\right)$ of *n* − 1 former stimulus observations. While *n* is the discrete time index of the consecutive trials, the value *N* denotes the total number of trials in a block within an experimental probability category for one subject. Note that in (1) stimulus *s*(*n*) has not yet been observed, therefore, a subjective probability distribution *P_k_*(*n*) for *all* possible stimuli *k* ∈ {1, …, *K*} on trial *n* is of interest. However, once the stimulus *k* = *s*(*n*) on trial *n* has been observed (which is only a *single* value *k* out of set {1, …, *K*}), the respective subjective probability *P_k_*(*n*) can be used to calculate the degree of *surprise* (Shannon and Weaver, 1948; Strange et al., 2005)

(2) $$I\left(n\right)=-\log_{2}P_{k=s\left(n\right)}\left(n\right).$$

Following Mars et al. (2008), we assume the trial-by-trial P300 estimate *Y*(*n*)[*μ*V] to be proportional to the surprise *I*(*n*)[bit]:

(3) $$Y\left(n\right)\propto I\left(n\right)$$

Note that Squires et al. (1976) assumed direct proportionality between the so-called *expectancy* *E_k_*(*n*) and the trial-by-trial P300 estimate:

(4) $$Y\left(n\right)\propto E_{k=s\left(n\right)}\left(n\right)$$

In the following we briefly recapitulate these two well-known state-of-the-art approaches to compute the subjective probability *P_k_*(*n*) or expectancy *E_k_*(*n*), which play the role of a *dynamically updated prior probability* for learning statistical parameters of the stimulus sequence.

#### 2.3.1 Approach by Mars et al. (MAR)

Mars et al. (2008) proposed a Bayesian observer model (henceforth called MAR) without forgetting according to

(5) $$P_{k}\left(n\right)=\frac{{\tilde{c}}_{\text{L,}k}\left(n\right)+1}{\left(n-1\right)+K},$$

where

(6) $${\tilde{C}}_{\text{L},\text{K}}\;\left(n\right)=\sum_{v=1}^{n-1}d_{k}\left(v\right)$$

counts the number of occurrences of event *k* until trial *n* − 1. The time sequence *d_k_*(ν) holds *d_k_*(ν) = 1 if *s*(ν) = *k*, ν = 1,2, …, otherwise *d_k_*(ν) = 0. Note that $\sum_{k=1}^{K}{\tilde{c}}_{\text{L},k}\left(n\right)=n-1.$ As can be easily seen in (5), the subjective probability for event *k* on trial *n* = 1 equals a uniform initial prior *P_k_*(1) = 1/*K*. After many trials (*n* ≫ *K*, and ${\tilde{c}}_{\text{L},k}\left(n\right)\gg 1$), the subjective probability approximates $P_{k}\left(n\right)\approx \frac{{\tilde{c}}_{\text{L},k}\left(n\right)}{n-1},$ i.e., the relative frequency of event *k* until trial *n* − 1. Note that the index “L” of the count function ${\tilde{c}}_{\text{L,}k}\left(n\right)$ expresses the *long*-*term memory* character of Mars’ model.

#### 2.3.2 Approach by Squires et al. (SQU)

Unlike Mars et al. (2008), Squires et al. (1976) did not formulate a strict computational model to compute the subjective probability *P_k_*(*n*). Moreover, having investigated solely a *K* = 2 case, they use the notion of *expectancy*^1^ *E_k_*(*n*) for stimulus *k* on trial *n*. While Squires et al. (1976) have described their model, hence called SQU, partly in math, partly in words, in the following we present a complete analytical formulation of their approach, which is straightforward to implement in software. Their empirical formulation of expectancy that event *k* ∈ {1, 2} will be observed on trial *n* ∈ {1, …, *N*} is given as

(7) $$E_{k}\left(n\right)=0.505.p_{k}+0.235.{\overset{⌣}{c}}_{s,k}\left(n\right)-0.033.{\overset{⌣}{c}}_{△,k}\left(n\right)-0.027$$

with three expectancy contributions, namely the assumed-to-be-known global probability *P_k_*, a count function for the *short*-*term memory* “S”, and a count function for the *alternation expectancy* “Δ” (and an additive constant).

##### 2.3.2.1 Short-term memory

The short-term count function is defined as

(8) $${\overset{⌣}{c}}_{s,k}\left(n\right)=\sum_{v=n-N_{\text{depth}}}^{\text{n}-1}\gamma_{\text{s}}^{\text{n}-v}d_{k}\left(v\right),$$

which is different to (6), since only a limited memory span of *N*_depth_ = 5 is covered, and an exponential forgetting factor $\gamma_{\text{S}}=e^{-\frac{1}{\beta_{\text{S}}}}$ with 0 ≤ γ_S_ ≤ 1 and time constant 0 ≤ β_S_ < ∞ is introduced, with γ_S_ = 0.6 for all probability categories (i.e., β_S_ = 1.96). Note that the count function (8) depends only on stimulus observations in the recent past.

##### 2.3.2.2 Global probability

The term *P_k_* = *P*(*s*(*n*) = *k*) in (7) denotes the true global probability of the stimulus being *k*. It is nothing else but the relative frequency of the stimulus in the current experimental probability category which must be made known to this model.

##### 2.3.2.3 Alternation expectancy

In contrast, the term ${\overset{⌣}{c}}_{\Delta ,k}\left(n\right)\in \left\{-3,-2,0,2,3\right\}$ denotes the expectancy w.r.t. alternating stimuli, and how this expectancy is met by the present stimulus *s*(*n*). The sign of ${\overset{}{c}}_{\Delta ,k}\left(n\right)$ is negative if the stimulus *s*(*n*) violates the alternation expectation [i.e., *s*(*n*) and *s*(*n* − 1) are identical] and positive if the alternation expectation is met [i.e., *s*(*n*) and *s*(*n* − 1) differ from each other]. The amplitude of ${\overset{}{c}}_{\Delta ,k}\left(n\right)$ depends on the number of *previous* alternations *in a row*. The formulas for calculating ${\overset{}{c}}_{\Delta ,k}\left(n\right)$ are detailed in the Appendix.

#### 2.3.3 Explanatory notes

In summary, (7) provides a model for expectancy that is linearly composed of three contributions: Firstly, the relative frequency *P_k_* of event *k* which equals the correct global probability throughout probability categories. Note that, in contrast to Mars et al. (2008), the relative frequency *P_k_* is not learned sequentially by experience but assumed to be known by participants. Secondly, a purely predictive limited length (*N*_depth_ = 5) exponentially decaying short-term memory [cf. count function ${\overset{}{c}}_{\text{S},k}\left(n\right)$ in (8)]. Thirdly, an expectancy contribution in the range $-3\;\leq \;{\overset{⌣}{c}}_{\Delta ,k}\left(n\right)\;\leq +\;3$ depending on the extent to which a first-order alternation (*aa* or *ba*) expectation has been build up and then met/violated within the latest observed *N*_depth_ = 5 trials.

### 2.4 Proposed digital filter model

**Figure 1:**
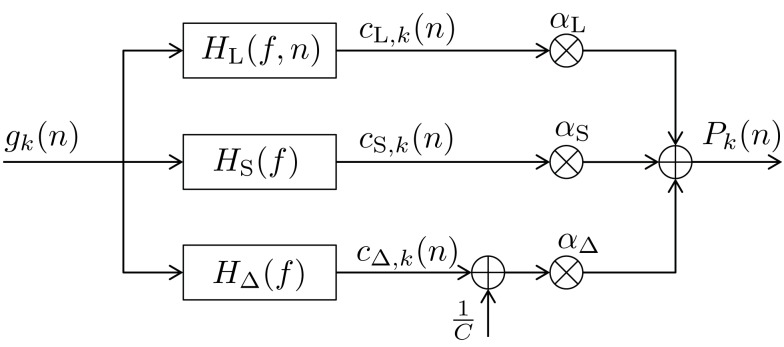
**Block diagram of the new digital filter (DIF) model with input *g_k_*(*n*) in (10) and output *P_k_*(*n*) in (9), digital filter transfer functions *H*(*f*) with 0 ≤ *f* ≤ *f_s_*/2, a stimulus presentation rate of *f_s_* (= 2/3 Hz), and probability normalizing constant 1/*C***.

In this section we present our newly proposed model, inspired by both Mars et al. (2008) and Squires et al. (1976). Our aim is to unify the learned relative frequency estimation of Mars et al. (2008; long-term) with the exponentially decaying short-term memory and alternation expectation capabilities of Squires et al. (1976), and to express the result in terms of a simple new *digital filter (DIF) model*. Besides an additive probability-normalizing constant 1/*C* (see Appendix for details), it consists of three additive contributions to subjective probability: a long-term contribution (“L”), a short-term one (“S”), and one term capturing alternations (“Δ”) as depicted in Figure 1:

(9) $$P_{k}\left(n\right)=\alpha_{\text{L}}\cdot c_{\text{L},k}\left(n\right)+\alpha_{\text{S}}\cdot c_{\text{S},k}\left(n\right)+\alpha_{\Delta }\cdot \left[c_{\Delta ,k}\left(n\right)+\frac{1}{C}\right].$$

There are three different count functions used, each represented by a digital filter transfer function *H*(*f* ) applied to the common input signal *g_k_*(*n*), which is given as:

(10) $$g_{k}\left(n\right)=\left\{\begin{array}{cc} \frac{1}{K},\; & \text{if}\;n\;\leq \;0\left(\text{uniform}\;\text{initial}\;\text{prior}\right)\\ 1,\; & \text{if}\;n>0\;\text{and}\;s\left(n\right)=k\\ 0,\; & \text{otherwise} \end{array}\right.$$

which implicitly contains an initial prior of 1/*K* at the start of a block of trials^2^, and a “1” wherever a past stimulus *s*(ν) equals the current stimulus *s*(*n*) = *k*, otherwise a “0.” Note that in contrast to the sequence *d_k_*(ν) as used in Mars et al. (2008) (6) and Squires et al. (1976) (8) we define a model-exciting infinite length *signal* *g_k_*(ν), ν ∈ {−∞, …, *n* − 2, *n* − 1}. The digital filter model yields an output signal *P_k_*(*n*) as given in (9). The weighting parameters α_L_, α_S_, α_Δ_ hold α_L_ + α_S_ + α_Δ_ = 1 and 0 ≤ α*_i_* ≤ 1, *i* ∈ {L,S,Δ}.

#### 2.4.1 Short-term memory

**Figure 2:**
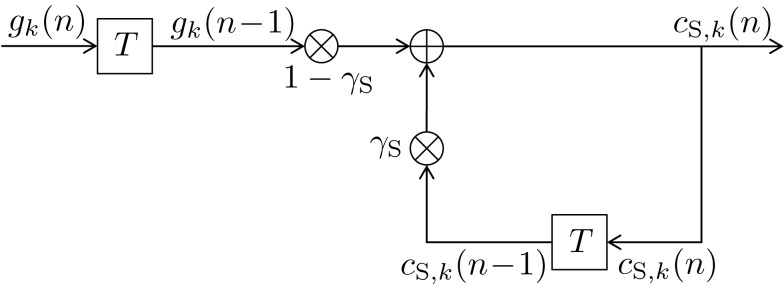
**Block diagram of a first-order infinite impulse response length (IIR) filter with transfer function *H*_S_(*f* ) equivalent to (11) or (12)**. Elements 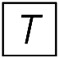 denote a delay of one trial. At the adder element ⊕, updating of the weighted output γ_S_·*c*_S,*k*_(*n* − 1) of the last trial *n* − 1 with the weighted input (1 − γ_S_)·*g_k_*(*n* − 1) of the last trial results in the current output *c*_S,*k*_(*n*). Note that via γ_S_·*c*_S,*k*_(*n* − 1), all preceding inputs (and outputs) influence the current output, though for the short-term memory, the influence of trials not in the recent past is negligible.

The block diagram of the infinite impulse response (IIR) digital filter is shown in Figure 2. The respective short-term memory count function can be expressed as

(11) $$c_{\text{S},k}\left(n\right)=\frac{1}{C_{\text{S}}}\overset{n-1}{\underset{\nu =-\infty }{\sum }}\;\gamma_{\text{S}}^{n-\nu }g_{k}\left(\nu \right),$$

with some normalizing constant *C*_S_ and an exponential forgetting factor $\gamma_{\text{S}}={\text{e}}^{-\frac{1}{\beta_{\text{S}}}}$ with 0 ≤ β_S_ < ∞, as with count function ${\overset{⌣}{c}}_{\text{s},k}\left(n\right)\;$ in (8). The transfer function of the short-term digital filtering process as described by (11) is depicted in Figure 1 as *H*_S_(*f* ), and is plotted in Figure 4 as dashed curve, revealing a smooth (i.e., weak) low-pass characteristic. Note that the short-term memory count function (11) can be expressed mathematically equivalent in a recursive form according to (see the Appendix)

(12) $$c_{\text{S},k}\left(n\right)=\left(1-\gamma_{\text{S}}\right)\cdot g_{k}\left(n-1\right)+\gamma_{\text{S}}\cdot c_{\text{S},k}\left(n-1\right),$$

initialized with the uniform initial prior *c*_S,*k*_(0) = 1/*K*, which in (11) was contained in the values *g_k_*(ν) = 1/*K* for ν ≤ 0 of (10). The recursive character of (12) becomes apparent by substituting the right hand side of (12) in itself for calculating *c*_S,*k*_(*n* − 1). Figure 2 further illustrates the updating process inherent in (12). The input signal defined in (10) and the weights (1 − γ_S_) and γ_S_ guarantee 0 ≤ *c*_S,*k*_(*n*) ≤ 1. The equivalence of (11) and (12) and the derivation of *C*_S_ = γ_S_/(1 − γ_S_) are shown in the Appendix.

#### 2.4.2 Long-term memory

The long-term memory count function can be expressed as

(13) $$c_{\text{L},k}\left(n\right)=\frac{1}{C_{\text{L},n}}\overset{n-1}{\underset{\nu =-\infty }{\sum }}\;\gamma_{\;\text{L},n}\left(\nu \right)g_{k}\left(\nu \right),$$

with the time-dependent (i.e., *dynamic*) exponential forgetting factor $\gamma_{\;\text{L},n}\left(\nu \right)=\prod_{\upsilon =\nu +1}^{n}\;\gamma_{\;\text{L},\upsilon }\frac{1-\gamma_{\;\text{L},\upsilon -1}}{1-\gamma_{\;\text{L},\upsilon }}$ $\left(\text{using}\;\gamma_{\;\text{L},\upsilon }=e^{-\frac{1}{\beta_{\text{L},\upsilon }}}\right),$ the dynamic normalizing value *C*_L,*n*_, and the same model-exciting signal *g_k_*(ν) as before (10). The formulas for calculating γ_L,*n*_(ν) and *C*_L,*n*_ are derived in the Appendix. The transfer function of the long-term digital filtering process as described by (13) is depicted in Figure 1 as *H*_L_(*f*,*n*). Analog to (12) a recursive function with the same behavior as (13) can be defined as

(14) $$c_{\text{L},k}\left(n\right)=\left(1-\gamma_{\;\text{L},n-1}\right)\cdot g_{k}\left(n-1\right)+\gamma_{\;\text{L},n-1}\cdot c_{\text{L},k}\left(n-1\right)$$

with the same initial value *C*_L,*k*_(0) = 1/*K*. The dynamics of the forgetting factor of the long-term memory are detailed in the Appendix.

**Figure 3:**
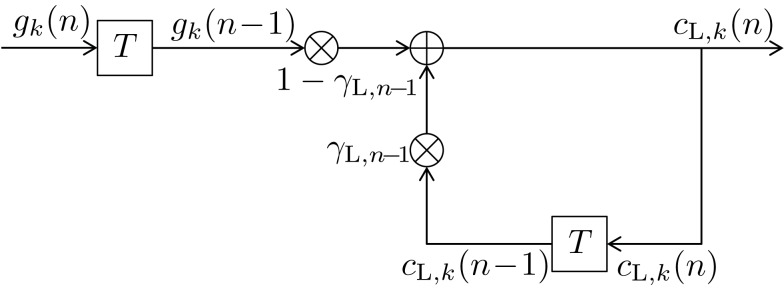
**Block diagram of a first-order infinite impulse response length (IIR) filter with transfer function *H*_L_(*f,n*) equivalent to (13) or (14)**. Elements 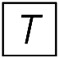 denote a delay of one trial. At the adder element ⊕, updating of the weighted output γ_L,*n*−1_·*c*_L,*k*_(*n* − 1) of the last trial *n* − 1 with the weighted input (1 − γ_L,*n*−1_)·*g_k_*(*n* − 1) of the last trial results in the current output *c*_L,*k*_(*n*). Note that via γ_L,*n*−1_·*c*_L,*k*_(*n* − 1), all preceding inputs (and outputs) influence the current output.

**Figure 4:**
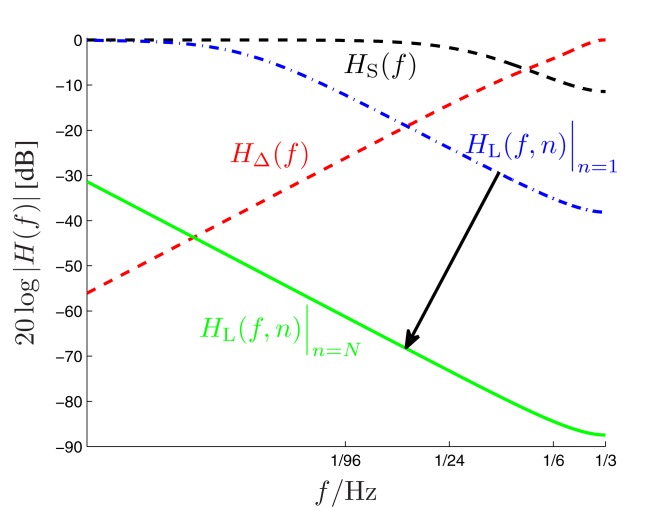
**Amplitude responses of the long-term (*H*_L_(*f*,*n*), dash-dotted and solid low-pass curves), short-term (*H*_S_(*f* ), dashed low-pass curve) and alternation (*H*_Δ_(*f* ), dashed high-pass curve) filters of the DIF model as a function of the input signal frequency *f* (logarithmic scale!)**. The dynamic long-term filter (dash-dotted and solid curves) is shown for *n* = 1 and *n* = *N* = 192.

Figure 3 illustrates the updating process inherent in (14). The long-term transfer function *H*_L_(*f*,*n*) is plotted in Figure 4 as dash-dotted curve (for *n* = 1) and as solid curve (for *n* = *N* = 192), respectively. Inspection of Figure 4 reveals an initially moderate low-pass characteristic which becomes much sharper when the number of trials increases.

#### 2.4.3 Alternation expectation

Finally, our model comprises a count function capturing alternations:

(15) $$c_{\Delta ,k}\left(n\right)=\frac{1}{C_{\Delta }}\overset{n-1}{\underset{\nu =n-4}{\sum }}\;\gamma_{\Delta ,n-\nu }\cdot g_{k}\left(\nu \right)$$

with some normalizing constant *C*_Δ_ and the same model-exciting signal *g_k_*(ν) as before (10). In contrast to the short- and long-term IIR filters which both have a low-pass characteristic, this finite impulse response (FIR) filter reveals a high-pass characteristic. Its transfer function is plotted in Figure 4 as ascending dashed curve. The coefficients γ_Δ,*n*−ν_ are specified in more detail in the Appendix.

#### 2.4.4 Explanatory notes

There are two important differences to Squires et al. (1976): We propose to use *two* terms with different time parameters β_S_ and β_L,*n*_, one accounting for the short-term memory [as in (8)], the other one accounting for a dynamically adapted long-term memory. Secondly, we allow for *N*_depth_ → ∞. Moreover, the role of negative trial indices in our model is to define the initial subjective probability distribution, which is *P_k_*(1) = 1/*K*, *k* ∈ {1, …, *K*} in (9), reflecting that participants were not informed about the actual relative frequencies of events over a block of trials.

In summary, the DIF model expresses both a long-term memory contribution, and a short-term memory contribution by exponential decay processes, with uniform initial subjective prior probabilities, and a contribution of alternation expectation. Though there are similarities between (7) and (9), the model of Squires et al. (1976) uses information about the experimental design (*P_k_*) which was actually unknown to the participants. In contrast, the DIF model uses only the information contained in the stimulus sequence as observed by participants, and it always starts with a uniform initial subjective probability distribution *P_k_*(1) = 1/*K*, *k* ∈ {1, …, *K*}, regardless of the actual relative frequencies of events over a block of trials. Finally it should be noted that our new model yields a conceptually well-defined subjective probability, as opposed to an expectancy as in Squires et al. (1976).

#### 2.4.5 Evaluation methods

Following Mars et al. (2008) we compared the DIF to the MAR and SQU models using the log-Bayes factor based on the model evidences. The evidences were approximated using the variational free energy which consists of an accuracy and complexity term, thus enabling the comparison and selection of competing models (Penny et al., 2004; Friston et al., 2007; Penny, 2012). We employed the same three-level hierarchical general linear model (GLM) as Mars et al. (2008). For model fitting and calculation of the model evidences we used parametric empirical Bayes (PEB) from the spm_PEB.m function of the Statistical Parametric Mapping (SPM8) software (Friston et al., 2002, 2007).

The different models (DIF, MAR, and SQU) generate the model-specific surprise^3^ *I*_ℓ_(*n*) or expectancy *E_k,ℓ_*(*n*) values as regressors, with the subscript ℓ ∈ {1, …, *L* = 16} denoting the individual participants and *n* ∈ {1, …, *N* = 192} being the discrete time index of the consecutive trials within one block. The first level of the GLM models the *measured trial*-*by*-*trial P300 estimates* *Y*_ℓ_(*n*) [μV] as a linear function of the surprise *I*_ℓ_(*n*) [bit] with the intercept $\theta_{\ell }^{\left(1\right)}\left[\mu \text{V}\right],$ the slope $\vartheta_{\ell }^{\left(1\right)}\;\left[\mu \text{V}∕\text{bit}\right],$ and an error $\epsilon_{n,\ell }^{\left(1\right)}\left[\mu \text{V}\right]:$

(16) $$Y_{\ell }\left(n\right)=\theta_{\ell }^{\left(1\right)}+\vartheta_{\ell }^{\left(1\right)}I_{\ell }\left(n\right)+\epsilon_{n,\ell }^{\left(1\right)}.$$

Note that the fitted model-based P300 estimates then follow

(17) $${\hat{Y}}_{\ell }\left(n\right)=\theta_{\ell }^{\left(1\right)}+\vartheta_{\ell }^{\left(1\right)}I_{\ell }\left(n\right).$$

The second level models the *participant*-*specific parameters* $\theta_{\ell }^{\left(1\right)}\;\text{and}\;\vartheta_{\ell }^{\left(1\right)}$ as *deviations from the corresponding group parameters* θ^(2)^ [μ*V*] and ϑ^(2)^[μ*V*/bit]:

(18) $$\begin{array}{rc} \theta_{\ell }^{\left(1\right)} & =\theta^{\left(2\right)}+\epsilon_{\theta ,\ell }^{\left(2\right)} \end{array}$$

(19) $$\begin{array}{rc} \vartheta_{\ell }^{\left(1\right)} & =\vartheta^{\left(2\right)}+\epsilon_{\vartheta ,\ell }^{\left(2\right)}. \end{array}$$

The third level functions as a shrinkage prior on the group parameters. In matrix notation the GLM structure is

(20) $$\begin{array}{c} \text{Y}={\text{X}}^{\left(1\right)}\Theta^{\left(1\right)}+E^{\left(1\right)}\\ \Theta^{\left(1\right)}={\text{X}}^{\left(2\right)}\Theta^{\left(2\right)}+{\text{E}}^{\left(2\right)}\\ \Theta^{\left(2\right)}={\text{X}}^{\left(3\right)}\Theta^{\left(3\right)}+{\text{E}}^{\left(3\right)}. \end{array}$$

**Y** = [**Y**_ℓ=1, …,_ **Y**_ℓ=*L*_]*^T^* ∈ ℝ^2*NL* × 1^ is a vector concatenating the trial-by-trial P300 estimates for all participants, with []*^T^* being the transpose, making **Y** a column vector. The participant-specific vector **Y**_ℓ_ = [*Y*_ℓ_(*n* = 1), …, *Y*_ℓ_(*n* = 2*N*)] ∈ ℝ^1 × 2*N*^ contains the trial-by-trial P300 estimates for one participant, averaged over the six sequence repetitions, for both probability categories. The first level design matrix **X**^(1)^ ∈ ℝ^2*NL* × 2*L*^ is block-diagonal with *L* partitions ${\text{X}}_{\ell }^{\left(1\right)}=\left[{\text{1}}_{2N}{\text{I}}_{\ell }\right]\;\in \;{\mathbb{R}}^{2N\times 2},$ each of which contains an all-one column vector **1**_2*N*_ of length 2*N*, and surprise values **I**_ℓ_ = [*I*_ℓ_(*n* = 1), …, *I*_ℓ_(*n* = 2*N*)]*^T^* ∈ ℝ^2*N* × 1^ as explanatory variables. The second level design matrix **X**^(2)^ = **1***_L_*⊗**I**_2 × 2_ = [**I**_2 × 2,ℓ=1, …_, **I**_2 × 2,ℓ=*L*_]*^T^* ∈ ℝ^2*L* × 2^ is the Kronecker product of an all-one column vector **1***_L_* of length *L* and an identity matrix **I**_2 × 2_. The third level design matrix **X**^(3)^ shall have all-zero elements. The unknown level-one parameters $\theta_{\ell }^{\left(1\right)}\;\text{and}\;\vartheta_{\ell }^{\left(1\right)}$ are assembled in the parameter vector $\Theta^{\left(1\right)}={\left[\theta_{\ell =1}^{\left(1\right)},\vartheta_{\ell =1}^{\left(1\right)},\ldots ,\theta_{\ell =L}^{\left(1\right)},\vartheta_{\ell =L}^{\left(1\right)}\right]}^{T}\in {\mathbb{R}}^{2L\times 1}$. Likewise, the second level parameters θ^(2)^ and ϑ^(2)^ are assembled in the vector Θ^(2)^ ∈ ℝ^2 × 1^. All errors are assumed to be normally distributed ${\text{E}}^{\left(j\right)}∼N\left(0,\Sigma_{\epsilon }^{\left(j\right)}\right).$ The covariance is parameterized following $\Sigma_{\epsilon }^{\left(j\right)}=\lambda^{\left(j\right)}{\text{I}}^{\left(j\right)}$, with **I**^(*j*)^ as an identity matrix with the same dimension as the number of rows of the design matrix of the corresponding level **X**^(*j*)^. The hyperparameters λ^(*j*)^ are the free parameters of the hierarchical linear model and are estimated using an EM algorithm for maximum likelihood estimation.

The conditional means of the first level parameters $\mu_{\Theta |Y}^{\left(1\right)}$ of the posterior densities $N\left(\mu_{\Theta |Y}^{\left(j\right)},\Sigma_{\Theta |Y}^{\left(j\right)}\right)$ were used as maximum *a posteriori* point estimates of the parameters for the model fitting for the Figures 6–8 and the calculation of the mean squared error (MSE) and fraction of variance explained (FVE) between ${Ŷ}_{\ell }\left(n\right)$ and Y_ℓ_(*n*) in Table 4 (Friston et al., 2002). The log-evidences or marginal log-likelihoods of the models *F* = ln(*p*(**Y** | ℳ)), with *p*(**Y** | ℳ) being the likelihood of the data *Y* given the model ℳ, were used for model comparison via the log-Bayes factor ln(BF) which is the natural logarithm of the quotient of the model likelihoods or the difference in log-evidence (Kass and Raftery, 1995; Penny et al., 2004; Friston et al., 2007):

(21) $$\ln\left(\text{B}{\text{F}}_{\text{DIF}-\text{XXX}}\right)=\ln\left(\frac{p\left(\text{Y}|M_{\text{DIF}}\right)}{p\left(\text{Y}|M_{\text{XXX}}\right)}\right)=F_{\text{DIF}}-F_{\text{XXX}}$$

If the log-evidence is calculated this way, positive values reflect evidence in favor of the DIF model and negative values in favor of the XXX model (being SQU or MAR), respectively. Values larger than five are considered “very strong” evidence (Kass and Raftery, 1995; Penny et al., 2004). To summarize, in our evaluation method we closely followed (Mars et al., 2008).

#### 2.4.6 DIF model parameter identification

The values for the free model parameters, namely α_L_ in (9), τ_1_ and τ_2_ (both (A12)), β_S_ [for (11)], α_Δ_ in (9), and γ_Δ,2_ in (15), have to be trained on the measured data, i.e., on the trial-by-trial P300 estimates. The model-based P300 estimates are calculated with the *same model parameters for all participants* and then used for maximization of the DIF model evidence, which is our optimization criterion, as described in Section 2.4.5.

The calculation of the model evidence for the whole range of possible combinations of the parameters with a reasonable resolution is computationally too expensive. For this reason only subsets of parameter combinations were optimized simultaneously with a resolution of 100 values per parameter, which results in 30,000 possible parameter combinations for one iteration. In the first iteration, while optimizing one set of parameters, the not yet optimized parameters were fixed to the center of their respective intervals. In the following iteration, parameters not currently optimized were fixed to the optimal values from the last iteration. In these two iterations a total of 60,000 parameter combinations have been evaluated, and the set with the highest evidence was considered optimal. Note that only a locally optimal parameter combination can be found using this procedure, as many iterations may be necessary for convergence toward the global optimum, if it can be found at all. Table 1 gives an overview over the searched parameter space.

**Table 1:** **The ranges of the free model parameters, with a resolution of 100 values per parameter**.

Parameter	Min	Max
α_L_	0.5	0.9
τ_1_	10	100
τ_2_	0.1	1
β_S_	1	10
α_Δ_	0.001	0.1
γ_Δ,2_	0.5	1

*Note that α_S_ is not a free parameter due to the restriction α_S_ = 1 − α_L_ − α_Δ_ and thus not optimized independently*.

## 3 Results

### 3.1 Behavioral results

RTs showed clear dependence on stimulus probability (0.3, *M* = 373.4 ms, *SE* = 6.9 ms; 0.5, *M* = 353.5 ms, *SE* = 7.7 ms; 0.7, *M* = 319.8 ms, *SE* = 6.4 ms). The slowdown of responding to less probable stimuli was confirmed by an ANOVA on RTs as a function of probability, *F*_(2,30)_ = 80.98, *p* < 0.001, $\eta_{p}^{2}=0.84,$ ∊ = 0.95. Polynomial contrasts revealed a linear trend, *F*_(1,15)_ = 166.79, *p* < 0.001, $\eta_{p}^{2}=0.92,$ in the absence of a quadratic trend, *F*_(1,15)_ = 3.37, *p* > 0.05.

Error rates similarly showed clear dependence on stimulus probability (0.3, *M* = 9.6%, *SE* = 1.6%; 0.5, *M* = 4.7%, *SE* = 0.9%; 0.7, *M* = 2.1%, *SE* = 0.4%). The enhanced error proneness in response to less probable stimuli was confirmed by an ANOVA on arcsin-transformed error rates as a function of probability, *F*_(2,30)_ = 25.19, *p* < 0.001, $\eta_{p}^{2}$ = 0.63, ∊ = 0.65. Polynomial contrasts revealed a linear trend, *F*_(1,15)_ = 28.94, *p* < 0.001, $\eta_{p}^{2}=0.66$ as well as a quadratic trend, *F*_(1,15)_ = 4.86, *p* < 0.05, $\eta_{p}^{2}=0.25$.

### 3.2 Conventional ERP results

**Figure 5:**
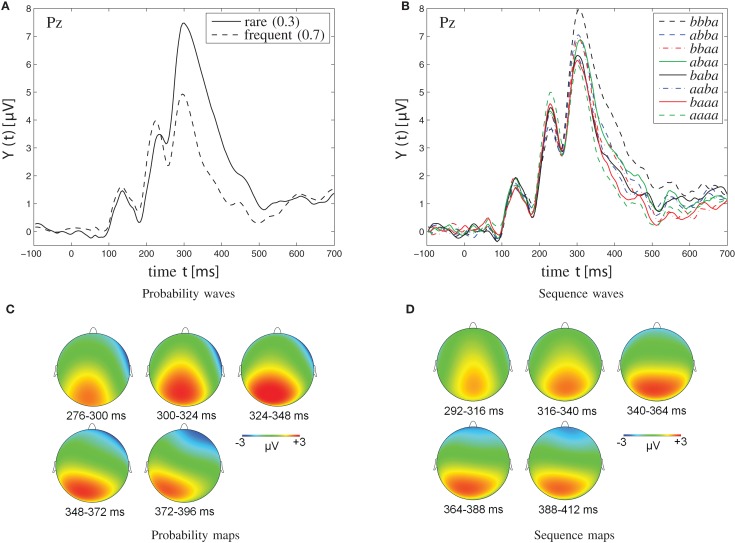
**Grand-average waveforms (A,B) and topographic maps (C,D) of P300 amplitudes**. **(A,C)** Probability effect on P300 amplitudes in the [0.3, 0.7] probability category. **(C)** The probability maps show the scalp topography of the rare-frequent difference wave in the [0.3, 0.7] probability category at various points in time (276–396 ms, divided into five windows of 24 ms each). **(B,D)** Sequence effect on P300 amplitudes in the [0.5, 0.5] probability category. Note that sequences of four successive stimuli are illustrated; *a* signifies a particular stimulus (*b* the other one). Note further that the two solid traces, originating from the *abaa* and the *baba* sequences, respectively, show reversed P300 amplitudes. Specifically, for the single -*b*- sequence *abaa*, the P300 waveform lies amongst those from dual -*bb*- sequences, whereas for the dual -*bb*- sequence *baba*, the P300 waveform appears indistinguishable from the waveforms from single -*b*- sequences. As further detailed in the Discussion, this amplitude reversal is attributed to the disconfirmation of alternation expectation in the *abaa* sequence, was well as to the confirmation of alternation expectation in the *baba* sequence. **(D)** Sequence maps show the scalp topography of the *bbba*-*aaaa* difference wave in the [0.5, 0.5] probability category at various points in time (292–412 ms, divided into five time windows of 24 ms each).

Figure 5 depicts grand-average ERP waveforms (upper panels) and topographic maps (lower panels). Left panels illustrate ERP waveforms at Pz that were obtained in the [0.3, 0.7] probability category. Right panels show third-order sequence effects on ERP waveforms at Pz that were obtained in the [0.5, 0.5] probability category. Note that sequences of four successive stimuli are illustrated, in temporal order (trial *n* − 3, trial *n* − 2, trial *n* − 1, trial *n* = eliciting event); *a* signifies a particular stimulus, *b* the other one. For example, *aaaa* gives a description of stimulus *a* being repeated across four consecutive trials (shown as green dashed curve), whereas *bbba* represents the presentation of stimulus *a* after having stimulus *b* repeated across the three immediately preceding trials (shown as black dashed curve).

As can be seen from Figure 5A, trial-by-trial P300 estimates showed clear dependence on stimulus probability (0.3, *M* = 4.84 μV, *SE* = 0.72 μV; 0.5, *M* = 3.51 μV, *SE* = 0.58 μV; 0.7, *M* = 2.00 μV, *SE* = 0.52 μV). P300 augmentation over stimulus improbability was confirmed by an ANOVA on P300 amplitudes as a function of probability, *F*_(2,30)_ = 39.88, *p* < 0.001, $\eta_{p}^{2}=0.73,$ ∊ = 0.82. Polynomial contrasts revealed a linear trend, *F*_(1,15)_ = 55.49, *p* < 0.001, $\eta_{p}^{2}=0.79,$ in the absence of a quadratic trend, *F*_(1,15)_ = 0.17, *p* > 0.05.

Sequential P300 estimates in the [0.5, 0.5] probability category (Figure 5B) yielded main effects of first-, *F*_(1,15)_ = 6.72, *p* < 0.05, $\eta_{p}^{2}=0.31,$ second-, *F*_(1,15)_ = 21.04, *p* < 0.001, $\eta_{p}^{2}=0.58,$ and third-order sequences, *F*_(1,15)_ = 6.89, *p* < 0.05, $\eta_{p}^{2}=0.32,$ as well as a significant three-way first- by second- by third-order sequence interaction, *F*_(1,15)_ = 6.70, *p* < 0.05, $\eta_{p}^{2}=0.31.$ First-order alternations (*ba*; *M* = 3.75 μV, *SE* = 0.57 μV) were associated with enhanced P300 amplitudes compared to first-order repetitions (*aa*; *M* = 3.26 μV, *SE* = 0.59 μV). Likewise, second-order alternations (*bxa*; *M* = 3.98 μV, *SE* = 0.64 μV) were associated with enhanced P300 amplitudes compared to second-order repetitions (*axa*; *M* = 3.03 μV, *SE* = 0.51 μV). Finally, third-order alternations (*bxxa*; *M* = 3.64 μV, *SE* = 0.58 μV) were associated with enhanced P300 amplitudes compared to third-order repetitions (*axxa*; *M* = 3.37 μV, *SE* = 0.57 μV). Separate ANOVAs on sequential P300 estimates were performed in each second-order sequence condition to further parse the three-way interaction. These ANOVAs revealed that the two-way first- by third-order sequence interaction was not significant when the second-order sequence consisted of stimulus repetitions (i.e., when *xaxa* sequences were included), *F*_(1,15)_ = 0.94, *p* > 0.05, whereas the two-way first- by third-order sequence interaction was significant when the second-order sequence consisted of stimulus alternations (i.e., when *xbxa* sequences were included), *F*_(1,15)_ = 6.34, *p* < 0.05, $\eta_{p}^{2}=0.30.$ Further comments on these data are deferred to the Discussion.

Sequential P300 estimates in the [0.3, 0.7] probability category yielded significant main effects of stimulus probability, *F*_(1,15)_ = 44.08, *p* < 0.001, $\eta_{p}^{2}=0.75$ (0.3, *M* = 4.59 μV, *SE* = 0.70 μV > 0.7, *M* = 2.20 μV, *SE* = 0.53 μV), of first-order sequence, *F*_(1,15)_ = 5.80, *p* < 0.05, $\eta_{p}^{2}=0.28$ (*ba*, *M* = 3.68 μV, *SE* = 0.58 μV > *aa*, *M* = 3.10 μV, *SE* = 0.63 μV), and of second-order sequence, *F*_(1,15)_ = 11.20, *p* < 0.01, $\eta_{p}^{2}=0.43$ (*bxa*, *M* = 3.81 μV, *SE* = 0.66 μV > *axa*, *M* = 2.98 μV, *SE* = 0.54 μV), but without interactions between these factors.

### 3.3 Model-based trial-by-trial analysis

**Figure 6:**
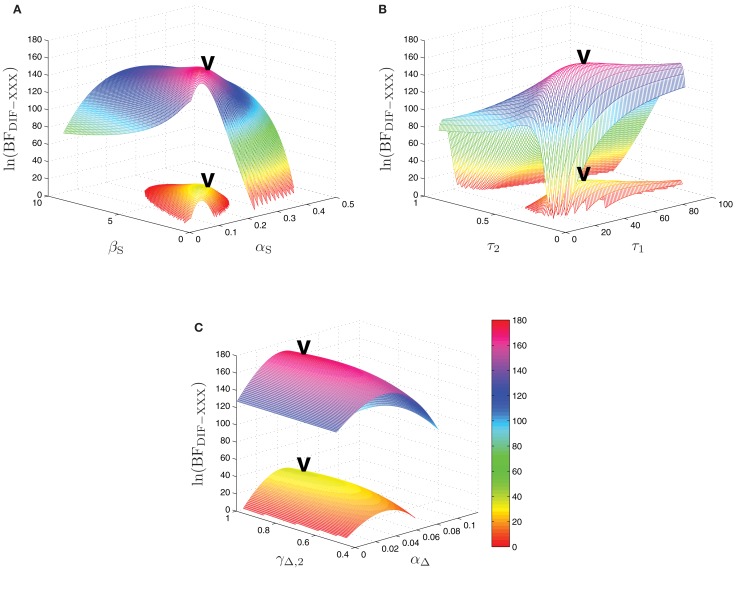
**Log-Bayes factors, ln(BF_DIF − XXX_), under variation of the model parameters**. The upper contour always shows ln(BF_DIF − MAR_), the lower one ln(BF_DIF − SQU_). The “V” marks the parameter combination with the maximum log-Bayes factor, cf. Table 3. **(A)** Variation of the free short-term parameters β_S_ and α_S_. **(B)** Variation of the free long-term parameters τ_1_ and τ_2_. In order to keep the lower contour visible, the log-Bayes factors for the 10 smallest values for τ_2_ are not displayed. **(C)** Variation of the free alternation parameters γ_Δ,2_ and α_Δ_.

**Figure A2:**
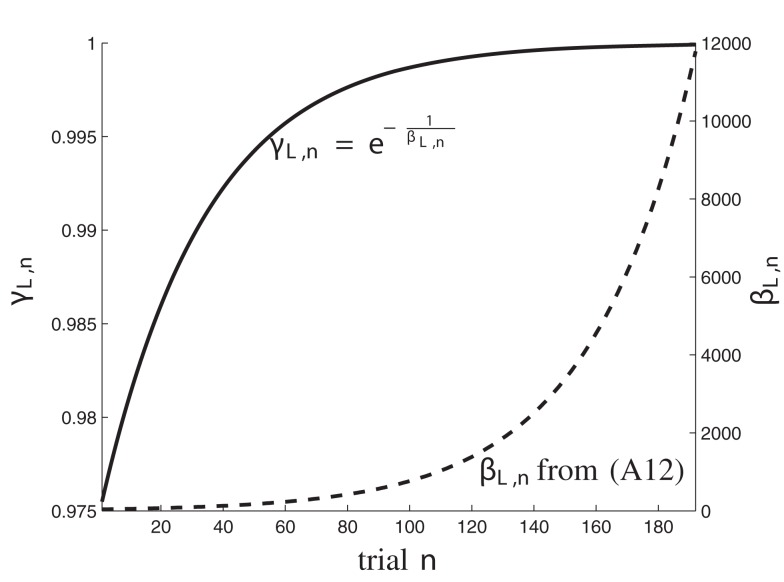
**The dynamics of the coefficients γ_L,*n*_ (solid) and *β*_L,*n*_ (dashed) over trials *n* = 1, …, *N*, with *N* = 192**.

The maximum log-Bayes factors in favor of the DIF model over the MAR and SQU models are shown in Table 2. In both cases, this is considered a *very* *strong* evidence in favor of the DIF model (Kass and Raftery, 1995; Penny et al., 2004). Table 3 shows the free parameters of the DIF model which were used for calculating the log-Bayes factors in Table 2. Figure 6 illustrates how the log-Bayes factors vary in dependence on the model parameters, with the tip of the “V” marking the parameter combination with the highest evidence. It is important to note the relatively flat tops of the contours implying good generalization capability of the DIF model. Due to computational complexity, only two parameters were optimized simultaneously, as described in Section 2.4.6. The relatively high value of α_L_ = 0.83 shows that the subjective probability mainly follows the long-term memory. With the identified values for τ_l_ and τ_2_ we get β_L,1_ = 40.3 and β_L,192_ = 11787, which is further illustrated in Figure A2 in the Appendix. With a short-term memory time constant of β_S_ = 1.82 and a weight of α_S_ = 0.12 the influence of recent events to the subjective probability is captured. While the weight of the filter modeling alternation expectancy α_Δ_ = 0.05 appears to be small, Figure 6C clearly shows the importance of this contribution.

**Table 2:** **The maximum log-Bayes factors ln(BF_DIF − XX__X_), left panel ln(BF_DIF − SQ__U_), right panel ln(BF_DIF − MA__R_)**.

ln(BF_DIF − SQU_)	ln(BF_DIF − MAR_)
35	170

**Table 3:** **The optimized model parameters**.

α_L_	τ_1_	τ_2_	α_S_	β_S_	α_Δ_	γ_Δ,2_
0.83	33.6	0.27	0.12	1.82	0.05	0.94

*Note that τ_1_ and τ_2_ yield β_L,1_ = 40.3 and β_L,192_ = 11787, respectively*.

**Figure 7:**
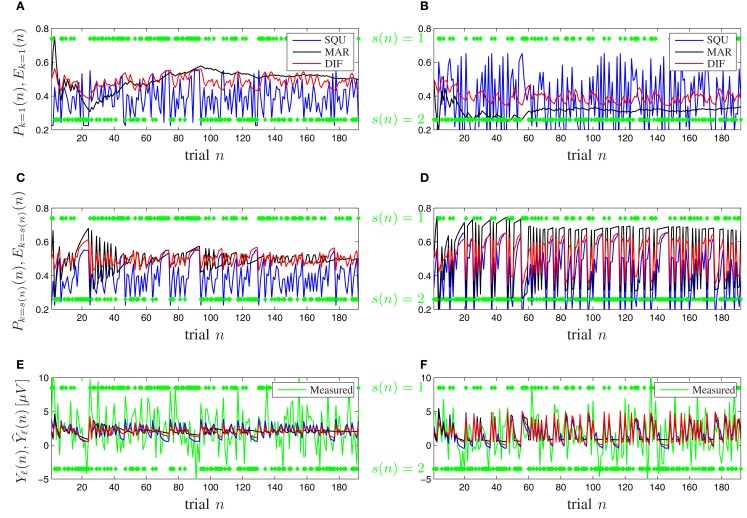
**Timeline plots for one exemplary participant**. **(A,C,E)** Probability category [0.5, 0.5]. **(B,D,F)** Probability category [0.3, 0.7]. Green symbols denote the stimuli *s*(*n*) ∈ {1,2} as they occurred. **(A,B)** Subjective probabilities *P*_*k*=1_(*n*) and expectancies *E*_*k*=1_(*n*) from (5), (7), and (9) for MAR, SQU, and DIF, respectively, for stimulus *s*(*n*) = 1. **(C,D)** Subjective probabilities *P*_*k*=s(*n*)_(*n*) and expectancies *E*_*k*=s(*n*)_(*n*) for the actually presented stimulus *k* = *s*(*n*). **(E,F)** The measured P300 estimates *Y*_ℓ_(*n*) and the model-based P300 estimates ${\hat{Y}}_{\ell }\left(n\right)$ of the MAR, SQU, and DIF models, respectively.

Figure 7 shows timeline plots for one exemplary participant. Figures 7A,B show plots of the expectancy *E*_*k*=1_(*n*) and the subjective probability *P*_*k*=1_(*n*) of seeing stimulus *k* = 1 on trial *n* for the [0.5, 0.5] and [0.3, 0.7] probability category for all competing models. Figures 7C,D show plots of the expectancy *E*_*k*=*s*(*n*)_(*n*) and the subjective probability *P*_*k*=*s*(*n*)_(*n*) of seeing *the actually occurring stimulus* *k* = *s*(*n*) on trial *n* for both probability categories and all models. The transition from Figures 7A,B to Figures 7C,D illustrates that the subjective probability is traced as a distribution for all possible events *k* ∈ {1, …, *K*} simultaneously over all trials and that at the moment of seeing a new stimulus *k* = *s*(*n*) only the corresponding subjective probability of that event *k* is relevant for the surprise *I*_ℓ_(*n*) and consequently for the model-based P300 estimate ${\hat{Y}}_{\ell }\left(n\right).$ Figures 7E,F show plots of both the measured and the model-based trial-by-trial P300 estimates *Y*_ℓ_(*n*) and ${\hat{Y}}_{\ell }\left(n\right)$ for both probability categories, where the latter is calculated according to (17). It is visible that the DIF model estimates are smoother over trials than those of the SQU model but not as undynamic as the estimates of the MAR model, which loses its initial dynamic and becomes almost binary over increasing trial number *n*. This effect is especially prominent in Figures 7E,F. Similar consecutive trials *n* elicit a descent in the measured trial-by-trial P300 estimate. For small *n* all models show this behavior, but for increasing *n* the MAR model yields nearly constant estimates.

**Figure 8:**
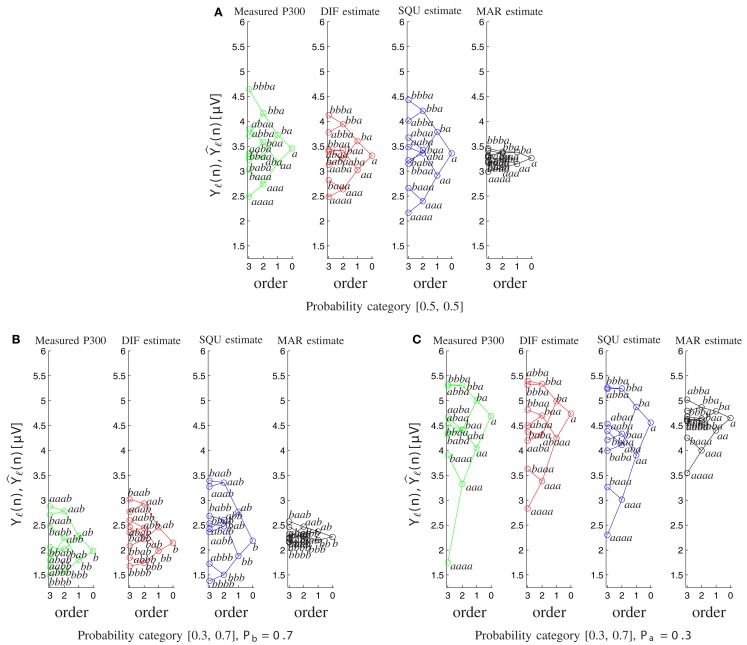
**Tree diagrams of measured P300 estimates *Y*_ℓ_(*n*) and model-based P300 estimates ${\hat{Y}}_{\ell }\left(n\right)$ as a function of the sequence of preceding stimuli**. Within each order (0–3), the stimulus sequence is labeled, and related sequences are connected by lines. **(A)** For both stimuli on probability category [0.5, 0.5]. **(B)** For the frequently occurring stimulus *b* on probability category [0.3, 0.7]. **(C)** For the rarely occurring stimulus *a* on probability category [0.3, 0.7].

Furthermore the MAR model does not account appropriately for the well-documented sequence effects (Squires et al., 1976). Figure 8 shows the tree diagrams of the measured (*Y*ℓ(*n*)) and model-based $\left({\hat{Y}}_{\ell }\left(n\right)\right)$ P300 estimates as a function of the preceding stimuli sequences for the different probability categories. The DIF and SQU model are both capable of estimating the envelope and general tree structure quite well, but the SQU model fans out too much for higher order effects for the frequent stimulus in the [0.3, 0.7] and in general in the [0.5, 0.5] probability category, while for the MAR model higher-order effects are nearly non-existent.

As an additional measure of goodness-of-fit of the models Table 4 shows the mean squared error (MSE) and the fraction of variance explained (FVE) of the fitted model predictions ${\hat{Y}}_{\ell }\left(n\right).$ Although differences appear to be somewhat smaller, still the superiority of the DIF model is evident, supporting the log-Bayes factors presented in Table 2.

**Table 4:** **Comparison of the goodness-of-fit in terms of the mean squared error (MSE) and fraction of variance explained (FVE) of the fitted model predictions ${Ŷ}_{\ell }\left(n\right)$**.

Model	MSE	FVE
MAR	6.0106	0.4909
SQU	5.7410	0.5138
DIF	5.6780	0.5191

## 4 Discussion

We tested three computational models of trial-by-trial P300 amplitudes using Bayesian model selection (Kass and Raftery, 1995; Raftery, 1995). Trial-by-trial P300 amplitude estimates at Pz were obtained in a two-choice RT task. Behavioral data indicated that on average participants reacted slower and committed more errors when they responded to rarely occurring stimuli, consistent with many earlier reports (Miller, 1998). P300 amplitudes showed the expected relationships with stimulus probability. Further, they were influenced by the immediately preceding stimulus sequence, a finding which is also consistent with earlier reports. Thus, our data replicate two of the most ubiquitous P300 findings, notably probabilistic and sequential effects on P300 amplitudes.

The DIF model (9) possesses important advantages over previous models of P300 amplitude fluctuations. It relies completely on mathematical notations and definitions, unlike the notions of expectancy (Squires et al., 1976), global vs. local probability (Squires et al., 1976), temporal probability (Gonsalvez and Polich, 2002), or context updating (Donchin and Coles, 1988). It is a formal model, akin to the MAR model (Mars et al., 2008), but it offers a more parsimonious explanation of trial-by-trial P300 fluctuations. The competitive advantage of the DIF model over the MAR model stems, in large part, from the non-negligible contribution of the short-term traces to subjective estimates of event probabilities, as evidenced by the scarce sensitivity of the MAR model to the sequential effects on P300 amplitudes (Figures 7 and 8).

The SQU model of P300 amplitude fluctuations (Squires et al., 1976) can be considered as a precursor of our DIF model insofar as it comprised memory for event frequencies within the prior stimulus sequence (equivalent to the short-term trace) and event probabilities (loosely related to the long-term trace). Yet, the long-term contribution to the DIF model does not incorporate global event probabilities, *P_k_*, themselves, but rather subjective estimates, *c*_L,*k*_(*n*), of these probabilities, these being based on counting observed events in continuously larger samples. Thus, subjective probability estimates are constantly revised while evidence is accumulating, and the DIF model is a pure model of subjective statistical parameters, rather than a mixture of subjective and objective parameters such as the SQU model.

Given the hereby documented superiority of the DIF model over its competitors, we will shortly consider some of its cornerstones. To begin with, it is important to view the DIF model in the context of the processing of event frequencies (Sedlmeier and Betsch, 2002). In particular, the reliable encoding of the frequency with which events occur (Underwood, 1969; Hintzman, 1976) led to the claim that event frequency is automatically encoded in memory, placing only minimal demands on attentional resources (Hasher and Zacks, 1984; Zacks and Hasher, 2002). A variety of representation modes for memory for event frequency can be envisaged. According to multiple-trace views (Hintzman, 1976), a record of individual events is stored such that each attended occurrence of an event results in an independent memory trace. In contrast, according to strength views, each attended event occurrence produces an increment in the strength of a single memory trace or a frequency counter (Alba et al., 1980), supporting the event frequency counter assumption which is inherent in the DIF model. Both, the short-term, *c*_S,*k*_(*n*), and the long-term, *c*_L,*k*_(*n*), memory traces are frequency counters (11) and (13).

The retention functions describing the short-term and long-term traces in the DIF model are of exponential-decay nature (Lu et al., 2005), differing mainly with regard to their decay half-lives. The dual decay rate assumption is compatible with the fact that short-term and long-term memory functions depend on dissociable neuronal processes (Jonides et al., 2008). Further, recent functional brain imaging data suggest different distributions of cortical responses for short-term and long-term decay functions (Harrison et al., 2011).

The optimal short-term time constant β_S_ approximated the value of two. Note that the DIF model does not allow for variations in β_S_, implying that the short-term contribution to the subjective probability for the appearance of event *k* on trial *n*, *P*_*k*=*s*(*n*)_(*n*), occurs stable over the progression of *n*. In contrast, β_L,*n*_ varies as a function of *n* (A12), with γ_L,*n*_ gradually approximating the value of one. β_L,*n*_ (and hence γ_L,*n*_) are relatively low during early trials when compared to late trials within blocks of trials (Figure A2 in Appendix). On the one hand, the long-term quality of the long-term contribution to *P*_*k*=*s*(*n*)_(*n*) is gradually increasing as a function of *n*, as revealed by the dynamics of the long-term low-pass filter (Figure 4). In other words, the decay half-life of the long-term trace gradually increases when the observer experiences more and more trials. On the other hand, the recursive formulation of the long-term contribution in the DIF model (14) reveals that the balance between the most recently experienced stimuli (which occurred on trial *n* − 1, weighted by (1 − γ_L,*n*−1_)) and the counted frequency (weighted by γ_L,*n*−1_) is biased toward recent stimuli during early trials (when γ_L,*n*−1_ is relatively low), but biased toward the counted frequency during late trials (when γ_L,*n*−1_ is relatively high). Thus, the DIF model postulates that the decay half-life of the long-term trace evolves dynamically with the amount of experience. The observer is modeled to rely more and more on environmental experience rather than on prior assumptions, possibly reflecting the fact that the exploitation of statistical redundancy becomes gradually more reliable with progressing time (Barlow, 2001).

Visual working memory (vWM, Baddeley, 2003) can maintain representations of only around four objects at any given moment (Cowan, 2005). The surprisingly limited vWM capacity offers a rationale for the assumption of a capacity-limited alternation term in the DIF model (15), *c*_Δ,*k*_(*n*). Its finite impulse response (FIR) characteristic resembles the alternation term in the SQU model (Squires et al., 1976). This FIR high-pass filter searches for alternation patterns over short sequences of trials (such as those in *abab* and in *baba* sequences). The discovery of such patterns leads one to expect the completion of the pattern in the upcoming trial, an expectation which will be confirmed in the *ababa* sequence, but will be disconfirmed in the *babaa* sequence. It is important that alternation expectation appears conditional upon the detection of alternation patterns in vWM, as revealed by the larger P300 amplitudes in response to *ba* sequences compared to *aa* sequences (Figure 8). Thus, the detection of alternation patterns in vWM entrains alternation expectation, as evidenced by our data (Figure 5B, see also Jentzsch and Sommer, 2001; Ford et al., 2010). Further, the effects of pattern completion (such as *baba* sequences) vs. pattern violation (such as *abaa* sequences) might underlie the first- by second- by third-order sequence interaction which we identified in the [0.5, 0.5] probability category.

While the effects of alternation expectation are non-negligibly measurable at Pz, visual inspection of our data at more anterior electrode sites suggested that these alternation expectancy effects might possess a more anterior, i.e., P3a-like scalp topography than the proper event frequency effects which showed the typical, P3b-like scalp topography (Polich, 2007; Duncan et al., 2009). While these initial observations ask for multi-channel data analyses, the present modeling work should be mainly considered as a model for P3b generation, since the task procedures (all stimuli required a button press) and the ERP waveforms [i.e., their scalp distribution (cf. Figure 5) and peak latencies] favor an interpretation in terms of predominant P3b-potentials.

**Figure 9:**
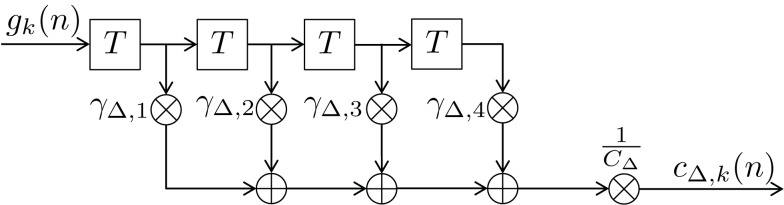
**Block diagram of the fourth-order finite impulse response (FIR) filter *H*_Δ_(*f* )**. Elements 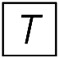 represent a delay of one trial, the multipliers γ_Δ,*i*_ compose the filter coefficients and *C*_Δ_ constitutes a normalizing constant.

The DIF model offers a digital filtering account of multiple memory systems in the brain (Figures 1–4). Specifically, the DIF model characterizes frequency memory as two digital first-order infinite impulse response (IIR) low-pass filters, one filter with an experience-invariant short-term exponential-decay function (Figure 2), and another filter with an experience-dependent long-term exponential-decay function, such that the low-pass characteristic becomes progressively apparent as the amount of experience increases (Figure 3). Moreover, vWM is conceptualized as an additional fourth-order finite impulse response (FIR) high-pass filter (Figure 9). The input signal *g_k_*(*n*) in (10) to all three filters is a binary representation of the stimulus sequence, with all samples prior to the first trial filled with the uniform initial prior.

Our theory of variation in trial-by-trial P300 amplitudes bears implications on the nature of cortical processing. It is in agreement with the predictive coding approach (Friston, 2002, 2005; Spratling, 2010). Viewed from this perspective, predictive surprise – and hence trial-by-trial P300 amplitude – is proportional to the residual error between top-down priors and bottom-up sensory evidence. Predictive coding theory is a successful guiding model for functional neuroimaging and electrophysiological studies of sensory cortical processing (Summerfield et al., 2006; Garrido et al., 2009; Summerfield and Egner, 2009; Egner et al., 2010; Rauss et al., 2011; Winkler and Czigler, 2011). Further, the DIF model is a Bayesian model of cortical processing (Knill and Pouget, 2004; Friston, 2005). It conceives performance on our two-choice RT task as sequential Bayesian learning (MacKay, 2003), with initial prior knowledge being conceptualized as a uniform prior probability distribution (10), consistent with Laplace’s Principle of Indifference.

It is important to note that we do not claim that the observed P300 modulations were exclusively related to predictive surprise *over* *sensory input*, since in the present task design the probabilities of sensory events were mirrored on probabilities of motor responses, as each stimulus was mapped onto a distinct motor response. Thus, particular stimuli also called for particular motor programs, and it could be that the observed P300 modulations are related to predictive surprise *over motor responses*. We deliberately leave it open whether the observed P300 modulations were due to surprise conveyed by the visual stimuli, or whether they were related to surprise associated with the selection of a motor response, given a visual stimulus on each trial (Barceló et al., 2008; O’Connell et al., 2012).

The DIF model specifies how predictive surprise determines trial-by-trial P300 amplitudes, seemingly representing barely more than a re-iteration of Donchin’s (1981) surprise hypothesis of P300 amplitude fluctuations. However, one should not confuse predictive surprise, as defined by the DIF model, with Bayesian surprise (Ostwald et al., 2012). Bayesian surprise numeralizes the divergence between *P*_1_(*n*), …, *P_K_*(*n*) and *P*_1_(*n* + 1), …, *P_K_*(*n* + 1), i.e., the divergence between probability distributions across successive trials which can be computed using the Kullback–Leibler metric (Baldi and Itti, 2010). Bayesian surprise thus quantifies the revision of the internal model of the world, given stimulus *s*(*n*), whereas predictive surprise *I*(*n*) in (2) refers to the unpredictability of *s*(*n*), given the internal model immediately before observing *s*(*n*). To conclude, we propose a formal computational model of predictive surprise, along with a strategy for testing the model’s ability to predict trial-by-trial P300 amplitudes.

## Conflict of Interest Statement

The authors declare that the research was conducted in the absence of any commercial or financial relationships that could be construed as a potential conflict of interest.

## Figures and Tables

**Figure A1:**
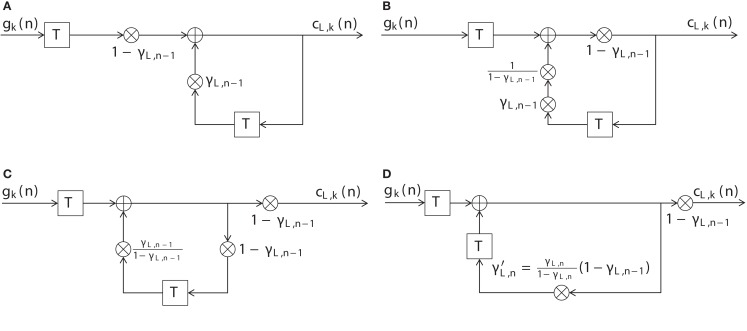
**Illustration of the equivalence of the digital filter (14) (Figure 3) to the count function as in (13)**. **(A)** Block diagram of the DIF model’s long-term memory filter of Figure 3 and (14) with input signal *g_k_*(*n*) and output signal *c*_L,k_(*n*). **(B)** Block diagram of a filter equivalent to **(A)**, where the multiplier (1 − γ_L,*n* − 1_) has been moved to the right. **(C)** Block diagram of a filter equivalent to **(B)**, where the multiplier (1 − γ_L,*n* − 1_) has been moved even further to the right. **(D)** Block diagram of a filter equivalent to **(C)**, where the multiplier γ_L,*n* − 1_/(1 − γ_L,*n* − 1_) has been moved to the right of the delay unit in the lower branch.

## Footnotes

^1^Squires et al. (1976) denote expectancy also as *subjective probability*.

^2^Participants were not informed about event probabilities, therefore a uniform initial prior distribution *P_k_*(1) = 1/*K*, *k* ∈ {1,…,*K*} is a reasonable model assumption before any stimulus has been observed.

^3^In the following, whenever *I*_ℓ_(*n*) is used, *E_k = *s*(*n*),ℓ_*(*n*) can be used interchangeably.

## Appendix

### Alternation expectation of the SQU model

This Appendix gives computational details on the expectation w.r.t. alternating stimuli ${\overset{⌣}{c}}_{\Delta ,k}\left(n\right)\;$ as required in (7) and described in words in Squires et al. (1976). The expectation w.r.t. alternating stimuli, and how this expectancy is met by the present stimulus *s*(*n*) is given as:

(A1) $$\begin{array}{l} {\overset{⌣}{c}}_{\Delta ,k}\left(n\right)\;=u_{k}\left(n\right)\cdot \left(\min\left\{N_{\text{alt}}-2,0\right\}+N_{\text{alt}}\right)\\ \;\in \left\{-\left(N_{\text{depth}}-2\right),\ldots ,-3,-2,0+2,+3,\ldots ,\left(N_{\text{depth}}-2\right)\right\} \end{array}$$

with *N*_depth_ = 5. The use of the minimum function ensures that an expectation for alternation requires at least *N*_alt_ = 2 consecutive previous stimulus alternations. Following Squires et al. (1976), its sign

(A2) $$u_{k}\left(n\right)=2\cdot |d_{k}\left(n\right)-d_{k}\left(n-1\right)|-1\in \left\{-1,+1\right\}$$

is negative (*u_k_*(*n*) = −1) if stimulus *s*(*n*) violates the alternation expectation [i.e., *s*(*n*) and *s*(*n* − 1) are identical]. On the other hand, if the alternation expectation is met [i.e., *s*(*n*) and *s*(*n* − 1) differ from each other], the sign is positive: *u_k_*(*n*) = +1. The number of *previous* alternations *in a row* constitutes the amplitude of the expectation, which is given by

(A3) $$N_{\text{alt}}=\arg\;\underset{N_{\text{alt}}^{\prime }\in N}{\max}\overset{N_{\text{alt}}^{\prime }}{\underset{\nu =1}{\prod }}\;\left[2\left|d_{k}\left(n-\nu \right)-d_{k}\left(n-\nu -1\right)\right|\right]$$

with^4^ $N=\left\{1,2,\ldots ,\left(N_{\text{depth}}-2\right)\right\}.$

### Alternation expectation of the DIF model

This Appendix gives details on the coefficients γ_Δ,*n*−ν_ and the probability normalizing constants *C* and C_Δ_. Figure 9 shows the block diagram of this fourth order finite impulse response filter. In order to reduce the complexity of the model by keeping the number of independent parameters small, only the coefficient γ_Δ,2_ is chosen freely within the range 0 ≤ γ_Δ,2_ ≤ γ_Δ,max_. The effect of the multiplicative constant *C*_Δ_ and additive constant *C* (as shown in Figure 1) is merely to ensure 0 ≤ *c*_Δ,*k*_(*n*) + 1/*C* ≤ 1. Thus, γ_Δ,max_ can be set to an arbitrary value if some constraints are met: The filter coefficients are set following γ_Δ,1_ = − γ_Δ,2_, γ_Δ,3_ = − γ_Δ,4_, and γ_Δ,4_ = γ_Δ,max_ − γ_Δ,2_. The normalizing constants have to be set according to: C_Δ_ = γ_Δ,max_ + γ_Δ,2_ + γ_Δ,4_ = 2γ_Δ,max_ and $C=\frac{\gamma_{\Delta ,\max}+\gamma_{\Delta ,2}+\gamma_{\Delta ,4}}{\gamma_{\Delta ,\max}}=2.$ We chose γ_Δ,max_ = 1.

### On count functions and digital filters (DIF model)

In this Appendix we present some mathematical details of the DIF model, its constants, and its relation to the count functions. We will show that the count functions (11) for *c*_S,*k*_(*n*) and (13) for *c*_L,*k*_(*n*) are equivalent to their recursive formulations (12) and (14), respectively. To this end we will transform the block diagram of Figure 3 to obtain the count functions from the new resulting block diagrams.

Figure A1A shows the block diagram of Figure 3 according to (14) with some notation omitted. A delay of one trial is represented by , the input signal is defined as *g_k_*(*n*), the output signal as *c_L,k_*(*n*), (1 − γ_L,*n*−1_) and γ_L,*n*−1_ are time-dependent values. The multiplier (1 − γ_L,*n*−1_) can be moved to the right yielding the block diagram shown in Figure A1B. Figure A1C shows the block diagram after moving (1 − γ_L,*n*−1_) even further to the right. Finally, when moving the multiplier γ_L,*n*−1_/(1 − γ_L,*n*−1_) to the right of the lower branch delay unit, the time dependency has to be accounted for as is shown in Figure A1D. For the long-term filter we obtain an effective filter coefficient

(A4) $$\gamma_{\;\text{L},n}^{\prime }=\gamma_{\;\text{L},n}\frac{1-\gamma_{\;\text{L},n-1}}{1-\gamma_{\;\text{L},n}}=e^{-\frac{1}{\beta_{\text{L},n}}}\frac{1-e^{-\frac{1}{\beta_{\text{L},n-1}}}}{1-e^{-1\beta_{\text{L},n}}},$$

while for the short-term filter this simplifies to

(A5) $$\gamma_{\text{S},n}^{\prime }=\gamma_{\text{S}}=e^{-\frac{1}{\beta_{\text{S}}}}.$$

The time-varying discrete-time impulse response of the long-term filter depicted in Figure A1D is given as

(A6) $$h_{\text{L},\nu }\left(n\right)=\epsilon \left(n-\nu -1\right)\frac{1-\gamma_{\;\text{L},n-1}}{\gamma_{\;\text{L},n}^{\prime }}\overset{n}{\underset{\upsilon =\nu +1}{\prod }}\;\gamma_{\;\text{L},\upsilon }^{\prime }$$

with *n*(>ν) being the currently observed trial, and ν being the trial when the stimulus initiated the impulse response. We make use of the step function

(A7) $$\epsilon \left(n-\nu -1\right)=\left\{\begin{array}{cc} 0,\; & n-\nu -1<0\\ 1,\; & n-\nu -1\geq 0.\\ \; \end{array}\right.$$

Substituting (A4) in (A6) yields

(A8) $$h_{\text{L},\nu }\left(n\right)=\epsilon \left(n-\nu -1\right)\frac{1-\gamma_{\;\text{L},n}}{\gamma_{\;\text{L},n}}\overset{n}{\underset{\upsilon =\nu +1}{\prod }}\;\gamma_{\;\text{L},\upsilon }^{\prime }=\epsilon \left(n-\nu -1\right)\frac{1}{C_{\text{L},n}}\overset{n}{\underset{\upsilon =\nu +1}{\prod }}\;\gamma_{\;\text{L},\upsilon }^{\prime }$$

with the dynamic normalizing value $C_{\text{L},n}=\frac{\gamma_{\;\text{L},n}}{1-\gamma_{\;\text{L},n}}.$ The input-output relation of this time-varying linear system is given by (c.f. Claasen and Mecklenbräuker, 1982; Prater and Loeffler, 1992):

(A9) $$c_{\text{L},k}\left(n\right)=\overset{\infty }{\underset{\nu =-\infty }{\sum }}\;h_{\text{L},\nu }\left(n\right)g_{k}\left(\nu \right).$$

Due to the step function (A7) in the impulse response (A6) only stimuli at trials ν ≤ *n* − 1 contribute to the output at trial *n* and we obtain

(A10) $$\begin{array}{ll} c_{\text{L},k}\left(n\right) & =\overset{n-1}{\underset{\nu =-\infty }{\sum }}\;h_{\text{L},\nu }\left(n\right)g_{k}\left(\nu \right)\\ & =\frac{1}{C_{\text{L},n}}\overset{n-1}{\underset{\nu =-\infty }{\sum }}\;\left(\overset{n}{\underset{\upsilon =\nu +1}{\prod }}\;\gamma_{\;\text{L},\upsilon }^{\prime }\right)g_{k}\left(\nu \right)\\ & =\frac{1}{C_{\text{L},n}}\overset{n-1}{\underset{\nu =-\infty }{\sum }}\;\gamma_{\;\text{L},n}\left(\nu \right)g_{k}\left(\nu \right), \end{array}$$

which is exactly the long-term count function (13) with $\gamma_{\;\text{L},n}\left(\nu \right)=\prod_{\upsilon =\nu +1}^{n}\;\gamma_{\;\text{L},\upsilon }^{\prime }=\prod_{\upsilon =\nu +1}^{n}\;\gamma_{\;\text{L},\upsilon }\frac{1-\gamma_{\;\text{L},\upsilon -1}}{1-\gamma_{\;\text{L},\upsilon }},$ and *g_k_*(ν), as before. Note that for the short-term filter we obtain $\prod_{\upsilon =\nu +1}^{n}\;\gamma_{\text{S}}=\gamma_{\text{S}}^{n-\nu }$ and *C*_S_ = γ_S_/(1 − γ_S_), which simplifies (A10) to the discrete-time convolution

(A11) $$c_{\text{S},k}\left(n\right)=\frac{1}{C_{\text{S}}}\overset{n-1}{\underset{\nu =-\infty }{\sum }}\;\gamma_{\text{S}}^{n-\nu }g_{k}\left(\nu \right),$$

which is the short-term count function (11). Thus, equivalence has been shown between the count functions (11) and (13), and their simple recursive formulations (12) and (14), respectively. The behavior of the dynamic long-term time value β_L,*n*_ follows:

(A12) $$\beta_{\text{L},n}=\left\{\begin{array}{cc} e^{-\left(\frac{1}{\tau_{1}}\cdot 1+\frac{1}{\tau_{2}}\right)},\; & \text{if}\;n\leq 0\\ e^{-\left(\frac{1}{\tau_{1}}\cdot n+\frac{1}{\tau_{2}}\right)},\; & \text{otherwise},\\ \; \end{array}\right.$$

with normalized time constants τ_1_, τ_2_, controlling the speed of transition from reliance on prior assumptions to experience. The time value 0 ≤ β_L,*n*_ < ∞ holds β_L,*n*_ > β_S_. The effect of this dynamic formulation of β_L,*n*_ is further illustrated in Figure A2 which shows the values of β_L,*n*_ and the corresponding forgetting factor $\gamma_{\;\text{L},n}=e^{-\frac{1}{\beta_{\text{L},n}}}$ for trials *n* ∈ {1, … *N*} with *N* = 192.
